# Prevention of gender-based violence in climate crises: entrenching feminist finance

**DOI:** 10.3389/fgwh.2025.1569482

**Published:** 2025-10-31

**Authors:** Vani Bhardwaj

**Affiliations:** Society of Gender Professionals, Frederick, MD, United States

**Keywords:** gender justice, climate justice, gender-based violence, prevention, feminist economics, forecast-based financing

## Abstract

The climate finance architecture addresses mitigation, adaptation, and loss and damage for climate-resilient development. However, it fails to advance the debt-related injustices in climate financing that inflict economic and non-economic violence on women from the low- and middle-income countries (LMICs) and marginalized communities in the “Global North.” Gender-based violence (GBV) is one dimension of climate injustices that becomes a risk multiplier to the lives of women, girls, and gender minorities across race, caste, abilities, and ethnicities. This article establishes the climate-resilient gender-responsive solutions to internalize gender-based violence prevention in the feminist economy of climate finance. Resultantly, the care economy under climate crises transforms family structures and relations beyond the neoclassical comprehension of micro and macroeconomics. This article takes a multisectoral approach to gender-responsive finance for climate crises. Moreover, it draws on gray literature from civil society organizations and think tanks addressing the majority of perspectives and academic articles across principles of feminist economics, climate financing, and gender-based violence.

## 1 Introduction

Care ethics, focusing on emotional awareness, synergize with sustainability transformations, displaying interconnectedness, context specificity, and intersubjective empowerment (1). Climate-related human trafficking and the misplaced emotive disposition against Women Environment Human Rights Defenders (WEHRDs) (2) in heteronormative patriarchal cultures create a hostile environment for gender just climate activism.

Caring for the environment entails caregiving to the surroundings, individuals, and communities, thus underlining a healthy balance between self-care and collective care (3). To address the feminist care economy, an ecosystem of gender data across formal and informal sectors to integrate labor-time saving technologies (4) is required. Feminist technologies can facilitate disaster risk reduction and prevent disaster impacts through gender transformative early monitoring systems that aid forecast-based financing (FbF) measures to prevent gender-based violence. Such feminist technologies that problematize and question masculinist technological norms by centering women's agency (5) can prevent climate-induced health insecurities.

Oftentimes, technology-facilitated gender-based violence (TF-GBV) places environmental activists in a precarious disposition, posing challenges for environmental cyber-activists across the gender spectrum compromising their cyber safety (6).

## 2 Health and wellness in anticipation of climate disasters: an intersectional lens

Trigger value at the lower end implies increased chances of false alarms. Assessing the trigger value for gender-based violence may be mapped and assessed qualitatively. Risks to GBV exposure can be encapsulated across qualitative indicators through Feminist Participatory Action Research (FPAR) methods (7). Albeit gathering the climate change-influenced school dropout rates and rates of child marriage, quantitative measurement of trigger thresholds for gender-based violence can remain equally challenging, particularly for household-level indicators in correlation to the likelihood of climate-related disasters.

Intra-household dynamics in families anticipating disasters in a perennial timeline can be motivated by “gendered adaptation behaviors” (8). Risk perceptions of gender-based violence fueled by climate injustice are influenced by traditional gender norms.

Garment sweatshops are dominated by women workers who require a safe workplace, and this becomes integral to climate mitigation and adaptation projects when it comes to sustainable clothing (9). Anticipatory actions informed by a trauma-informed approach could result in effective anticipatory strategies, such as a lens that includes a crosscutting outlook on the determinants of climate change-induced displacement due to recurrent climate disasters, given that more than half the refugee population is constituted by women with their children (10). Economic abuse has a positive correlation with varying intensity for different forms of gender-based violence (11). Economic neglect, economic sabotage, and economic displacement due to climate-related events and risks along with sexist cultural norms (12, 13) conflate to form an ecosystem of climate change-driven economic abuse along gendered lines.

The anticipation of violence enmeshed in trust deficit across the community stakeholders, whether in climate risks or in everyday sexism and racism, creates an atmosphere of fear and eco-anxiety. The root causes of such trust deficit are determined by information asymmetry (14) and tepid environmental communication to the masses. The role of civil society organizations in building a trust culture in an ecosystem of impact-based forecasting or anticipation of displacement-related violence becomes critical to examine (15). In the context of climate change, adaptive mechanisms seem coherent to mobility that may highlight gendered experiences (16, 17).

Pre-trauma or pre-traumatic stress syndrome (PreTSS) regarding climate change is proliferating across the public arena from popular culture to media (18). However, examination of the class context of the nature of pre-trauma related to climate catastrophic events needs to be conducted. For instance, would a family anticipating flooding of their informal settlement every year have a higher intensity of anticipatory trauma, or would a daily wage laborer on a construction site be more apprehensive of heat waves in urban areas, or a housing society middle-class resident be more wary of the future climate catastrophes? AI-driven climate models that inform early warning systems for disaster monitoring are ill-equipped to capture the socio-emotions of such communities in the low- and middle-income countries (LMICs), and the magnitude of such socio-emotional consequences of climate-related disasters across intersectionally identified populations reflects the hyper-masculinist nature of AI models. Take, for example, examining how anticipatory climate trauma varies in quality and intensity between the elderly and the youth from different races, abilities, and socio-cultural contexts.

Inability to find solace in oneself with the state of the environment as it degrades with changing landscape, as environmental disasters surmount, and climate injustice picks up pace, is labeled as solastalgia (19, 20). Samantha Stanley elaborates on the “future oriented mourning and distress” as a component of anticipatory solastalgia (21). Anticipatory solastalgia takes place in the present from a future perspective, while anticipated solastalgia is related to environmental risks that may take place somewhere in the future but not presently (22). Decolonial epistemes inform solastalgia, and an indigenist perspective is required to comprehend solastalgia for the indigenous population. Upward et al. (23) use the Aboriginal Participatory Action Research (APAR) as a method to assert that the loss of indigenous cultural wisdom becomes an intergenerational loss that contributes to the deterioration of the socio-ecological system for the indigenous and the non-indigenous population.

In contrast to solastalgia, climate anxiety pertains to personal trepidations regarding climate change and its concomitant injustices (24). Exposure to social media, occupational profile of parents, and class stratification can be potential indicators to assess eco-anxiety (25). However, studies establishing a correlation of queer beingness to eco-anxiety, eco-grief, solastalgia, climate trauma, and climate paralysis have not been undertaken from non-binary perspectives.

Women have been reported to display higher levels of eco-anxiety. Heeren et al. (26) attribute the positive correlation between gender and eco-anxiety to the innate emotional self-awareness within women as compared with men. However, such analysis entails essentialization of gender performativities and ignores the complex emotional beings that individuals become in their situatedness. Climate change can trigger systemic instability and insecurities due to the “climate chaos” that brings transformations for the poor, homeless, internally displaced, and urban communities (27).

Socio-emotional anxiety in relation to climate change has been measured across distinct indicators such as environmentally affirmative behaviors, political inclinations, and policy proclivities (28). However, a lack of gender disaggregated analysis across different communities in terms of crosscutting marginalization is desirable for extrapolating more in-depth drivers of eco-anxiety.

## 3 Gender in adaptation finance adherence to the prevention of GBV principles

There is no universally concurred nomenclature of what “climate finance” entails; however, financial resources mobilized for mitigation, adaptation, and managing consequences of climate change fall under the domain of climate finance mechanisms under United Nations Framework Convention on Climate Change (UNFCCC) (29). A lifecycle-based approach to climate financing by cohering Women, Children, Adolescent Health (WCAH) financing mechanisms with climate justice ensures that climate adaptation and mitigation finance become gender responsive on a continuum for the underserved communities (30).

Infrastructure management for resilient adaptations to take hold is essential, while poor emergency handling of infrastructure is one of the main contributors to limits to adaptation (31). Legal lacunae, policy gaps, weak regulations, and a lack of political will can inhibit the mobilization and allocation of decentralized climate finance (32).

GBV prevention principles emphasize survivor-centric principles, women's empowerment for creating safe spaces, risk mitigation, legal assistance, psychosocial support, and case management for GBV survivors, monitoring, evaluation, and learning (33).

Humanitarian environments function under the Inter-Agency Standing Committee (IASC) protection from sexual exploitation and abuse (PSEA) guidelines that prevent any sexual abuse and sexual harassment by humanitarian aid workers (34). However, since responses to the climate crisis fall outside the purview of humanitarian aid, women and girls vulnerable to climate fragilities are not covered under PSEA guidelines and therefore are unsafe.

Sendai Framework Gender Action Plan discusses “localized approach to anticipatory action” (35) wherein policymakers should decentralize foresight analytics to local contexts of women across fragile settings and disaster-prone geographies. Therefore, involving local women in developing their own anticipatory action plans synergizes with forecast-based financing (FbF) for GBV prevention.

Impact-based forecasting leading to forecast-based financing needs to avoid maladaptation, in case forecasting mechanisms harm GBV prevention principles. For instance, adaptation finance for local projects that cater to minor forest produce government schemes and plantations might overlook the long-distance mobility that often women of the household have to undertake to collect minor forest produce. It puts these women in dangerous contexts and vulnerable to harassment and other forms of violence as they traverse dense forests or plantations (36, 37). Such circumstances are reflective of maladaptation in the long term, even as women-inclusive employment seemingly becomes addressed.

As prevention of GBV principles fail, the limits of adaptation are reached for women and gender minorities. Losses and damages anticipated for a community need to recognize the monetary and non-monetary ramifications of maladaptation. The Loss and Damage Fund, while addressing maladaptation, should also be aligned with FbF to target the prevention of gender-based violence triggered by climate-induced disasters (38).

Coping mechanisms to tackle climate anxiety can become maladaptive if addressed person-to-person rather than systemically (39), such as through community health programs and public health campaigns to enhance prevention of sexual abuse and exploitation.

## 4 Discussion

### 4.1 Forecast-based financing (FbF) should synergize with principles of gender-based violence prevention

Climate finance enhances the resilience of socio-ecological systems (40) and must be turned feminist to cater to women's sense of safety during climate-fueled crises across communities. The anticipatory climate trauma endured by women due to possibilities of climate disasters, particularly the rapid onset ones such as the floods and cyclones, can be prevented if institutions are strengthened for gender-based violence prevention. The climate finance narrative, while evidently fixated on historical injustices, is in fact *also* driven by an anticipatory fear of the historical injustices getting replayed.

To achieve the climate targets by 2050, high-income countries owe a compensation of US$192 trillion to LMICs (41). The reparation climate economy, however, misses out on non-financial structural damages caused, quantifying which can amount to erroneous nomenclature. Will the compensatory climate finance be sufficient to reverse the losses and damages caused to the economies of LMICs? Firstly, compensatory climate economy does not address the racial and other forms of systemic violence inflicted by climate injustices, such as psychosocial consequences due to climate-related gender-based violence and climate-induced displacement. Secondly, the compensatory approach implies that the climate funding will only operate in an ex post manner, rather than for preventive and ex ante purposes. The binary aspects to “Global North” and Majority World stand challenged as peripheral aspects to the former are also crucial to ensure an intersectional analysis.

Usually, there are numerous thresholds driven by time-differentiated forecast probabilities. A summation of FbF depends on the sequence of forecast probabilities across time. The amount disbursed needs to be proportional to the probability of the occurrence of a disaster to be cautious of false alarms. The forecast-based financing must be measured by the twin objectives of effectiveness of anticipatory action and likelihood of disaster occurrence (42).

**Figure 1:**
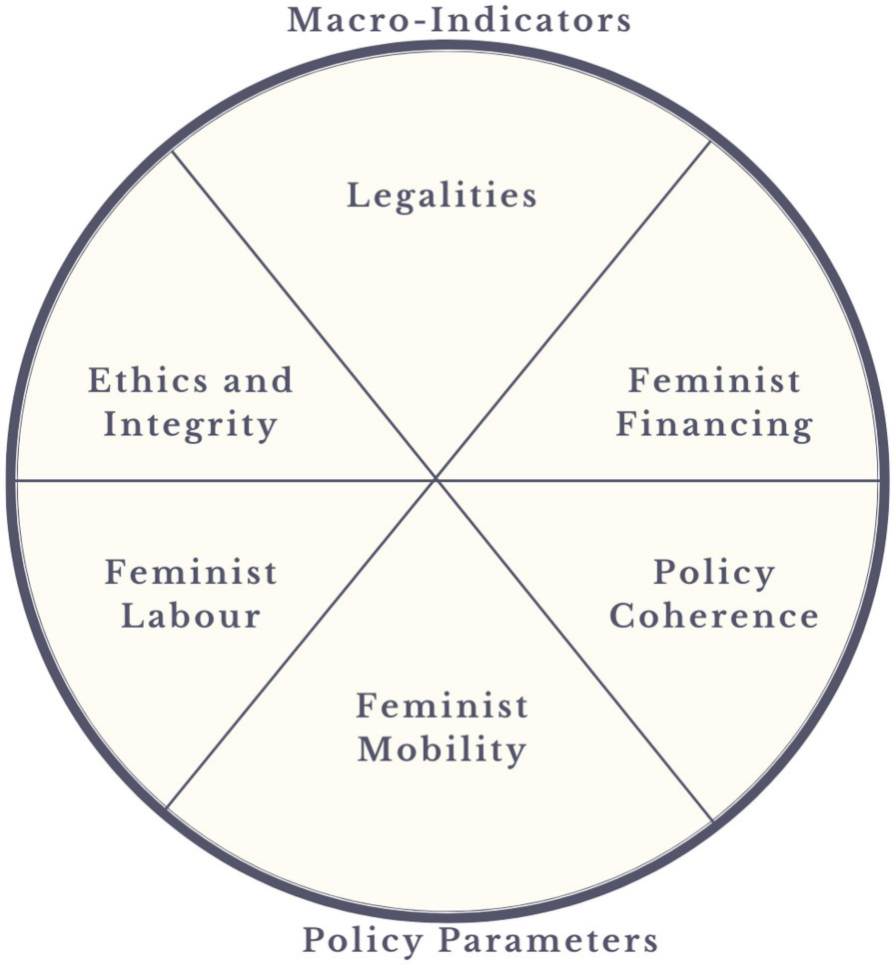
Macro-indicators interact with policy parameters for disaster risk reduction projects to consolidate anticipatory feminist based financing (FbF).

The gender-based violence exacerbated by climate-related disasters requires gender determined indicators (See Figure 1) to determine anticipatory trigger thresholds. The triggers for gender-based violence influenced by climate injustices are a mix of contextual and structural oppression (43). FbF should be determined by gender-based violence triggers getting activated by the likelihood of climate-induced disasters. Once the anticipatory funds are disbursed, the monitoring and accountability of matching the trigger thresholds for gender-based violence involves multi-stakeholder rapid gender analysis.

FbF narratives are built around anticipated material losses and damages such as anticipating loss of lives and infrastructure by probable disasters. While the literature discusses false alarms and misallocation of FbF, it remains focused on disaster prevention in terms of droughts and cyclones but never takes into account the unintended “costs” of the implicit disasters or crises of gender-based violence, ranging from homophobic and transphobic violence to violence against women and girls. Neither are the psychosocial well-being aspects fueled by climate trauma and eco-anxiety taken as indicators in forecast-based financing. The emphasis remains on insurance schemes and food security schemes under the FbF operations (44).

**Figure 2:**
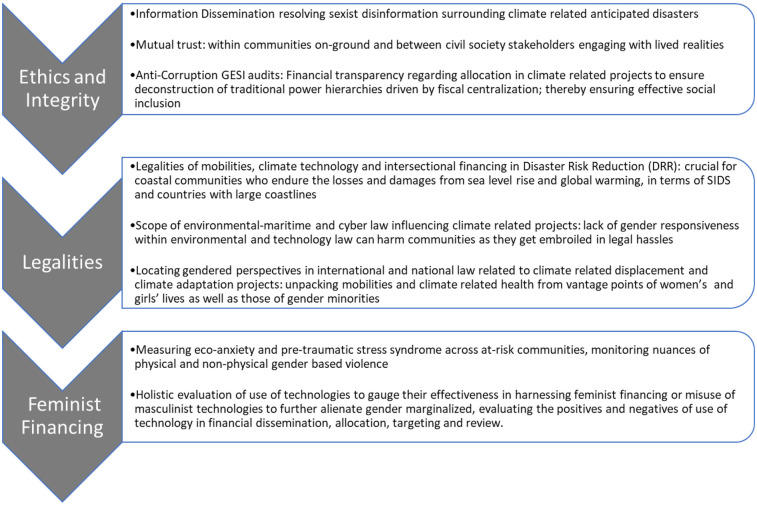
Macro-indicators for anticipatory FbF of disaster risk reduction (DRR)-related climate projects.

However, FbF should also be targeted for the prevention of intimate partner violence, domestic violence, and prevention of sexual harassment in mobility in disaster-prone regions. The vulnerability mapping of such climate-fragile geographies must be undertaken by rapid gender analysis (45) that aligns with a feminist care approach for allocation of FbF (See Figure 2).

## 5 Dual approach to FbF for prevention of GBV in the context of climate risks

This requires women-centric designing of angel investing such as networks of women angel investors (46) for serving the underserved and unserved communities in a gender transformative manner. Women in gender-lens investing in health equity and climate justice require that the prevention of GBV should be enhanced. Women leaders are involved in financing gender-based violence prevention, and a women-centric design of feminist financing ensures prioritization of gender-responsive issues across civil society organizations and humanitarian contexts (47, 48).

Convergences across principles of feminist economics, vis-à-vis care economy and social solidarity economy principles of decentralized empowerment of the marginalized have become evident (49). Feminist Social Solidarity Economy (FSSE) can be applied in contexts of grassroots collectives handling climate injustices and mainstreaming and transforming climate narratives (50).

Both these models of gender-responsive financing entail a multisectoral approach to ensure prevention of gender-based violence due to exacerbated climate risks, as per the contexts that prevail in each sector, whether as a social enterprise or a social solidarity collective.

**Figure 3:**
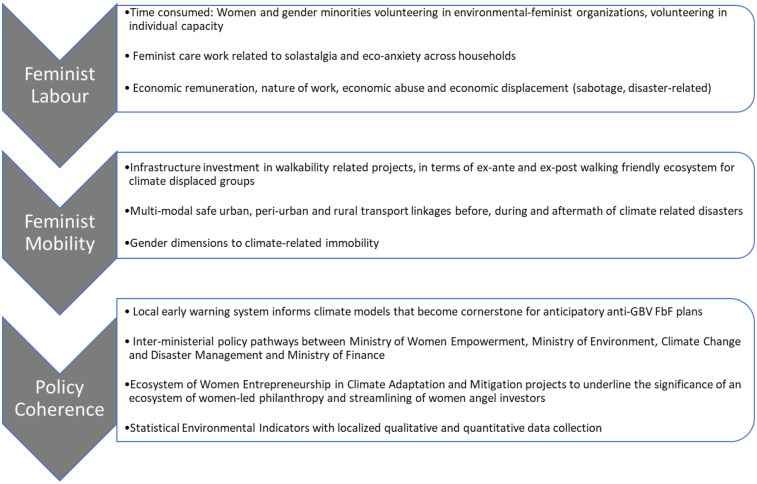
Policy parameters for anticipatory FbF of DRR-related climate projects.

This article has encapsulated care ethics in the context of feminist economics to analyze the gender-responsive nature of forecast-based financing in the context of climate risks and disasters. It also has discussed health inequities, the ambiguity in deciding risk thresholds, and maladaptation to feminist climate financing. The prevention of gender-based violence, as a priority lens for such FbF principles, should thereby be entrenched in feminist, women-led agency and leadership in feminist financing to ensure gendered climate justice. The queering aspects to FbF in enhanced risks to gender-based violence and its prevention are an understudied niche and need to be developed further. Lastly, this article provides macro-indicators and policy parameters, extrapolated from the literature reviewed, for disaster risk reduction projects from queer-feminist lenses that can be adopted by civil society organizations and other stakeholders (See Figure 3).

## Data Availability

The original contributions presented in the study are included in the article, further inquiries can be directed to the corresponding author.
